# A multiattribute group decision-making method based on a new aggregation operator and the means and variances of interval-valued intuitionistic fuzzy values

**DOI:** 10.1038/s41598-022-27103-z

**Published:** 2022-12-29

**Authors:** Ruipu Yao, Huijuan Guo

**Affiliations:** School of Information Engineering, Tianjin University of Commerce, Tianjin, 300134 China

**Keywords:** Applied mathematics, Computational science

## Abstract

The development of information measures associated with interval-valued intuitionistic fuzzy values (IVIFVs) has been an important research area over the past few decades. In the literature, the existing decision -making method using IVIFVs has some drawbacks, and the identification degree and information utilization suffer from a gap in the evaluation of alternatives. Therefore, the need for a reliable, useful, and comprehensive decision method is obvious. To obtain more accurate and reliable evaluation results, multiattribute group decision-making (MAGDM) problems, where the same attribute weights given by different decision-makers are different, are studied in this paper. First, the novel operational laws of IVIFVs and a new interval-valued intuitionistic fuzzy weighted arithmetic aggregation operator are defined to overcome the drawbacks of the IIFWA aggregation operator and avoid losing or distorting the original decision information in the process of aggregation. Second, the mean and variance of the possibility degrees of IVIFVs are defined based on the concept of a definite integral. Third, a novel MAGDM method based on the new aggregation operator and the mean and variance of the possibility degrees of IVIFVs is proposed to improve the identification of the evaluation results and ensure the effectiveness of the ranking order. Finally, the effectiveness and practicability of the proposed method are verified by an air combat training accuracy assessment example. This example can be used to assist decision-makers in evaluating air combat training hits in a timely and efficient manner, providing an objective, scientific basis for the realization and application of air combat training hit assessment and a new method and idea for MAGDM problems in an interval-valued intuitionistic fuzzy environment.

## Introduction

Due to the uncertainty and fuzziness of decision-making problems with uncertain information, it is difficult for decision-makers to sort and select alternatives. Zadeh^1^ successively proposed the theory of fuzzy sets and the concept of linguistic variables^2–4^ in 1965 and 1975, respectively. Furthermore, Atanassov^5^ defined the concept of intuitionistic fuzzy sets (IFSs), which are a strong tool used to address hesitation and uncertainty. IFSs have been widely applied in multiattribute decision-making (MADM)^6–9,11,13–15^. To overcome the shortcomings of IFSs, Atanassov and Gargov^16^ proposed the concept of interval-valued intuitionistic fuzzy sets (IVIFSs). Because intervals are adopted in IVIFSs to represent the membership degree, nonmembership degree and hesitation degree information, they are more flexible and practical than IFSs in dealing with fuzziness and uncertainty^17^. In recent years, IVIFSs have been widely applied in multiattribute decision-making (MADM)^10^, and many researchers have investigated the theories and methods of IVIFVs and applied them to the field of MAGDM problems. In applications of IVIFVs, how to evaluate and rank alternatives accurately and choose the optimal alternative is the key to addressing the MAGDM problem. Zhao et al.^12^ extended the CPT-TODIM method for interval-valued intuitionistic fuzzy MAGDM and applied it to urban ecological risk assessment. You et al.^18^ proposed a novel MAGDM method based on the correlation coefficient and hesitancy degree in an interval-valued intuitionistic fuzzy environment. They used the TOPSIS and linear programming optimization methods to calculate the optimal attribute weight and obtain more accurate weights. Ye^19^ proposed an MADM method based on an extended TOPSIS method with IVIFVs. Chen X et al.^20^ introduced a method to construct a class of distance measures for the intuitionistic hesitant fuzzy set and proposed an extended intuitionistic hesitant fuzzy technique for order preference by similarity to an ideal solution, i.e., the TOPSIS method. With the technique for order preference by similarity to the TOPSIS method, Hou et al.^21^ built a group decision-making model. Liu and Jiang^22^ proposed an MADM method by defining a new distance measure for IVIFVs. Garg and Kumar^23^ transformed IVIFVs to connection numbers in set pair analysis theory and proposed a novel MADM method. By using interval-valued intuitionistic fuzzy preference relations, Zhang and Chen^24^ proposed a method to solve the priority level of IVIFVs and then proposed a group decision-making method based on optimization.

Another active research topic is the investigation of MAGDM by introducing intuitionistic fuzzy aggregation operators. Atanassov^25^ defined the basic operational laws of IVIFVs, and Xu and Yager^26^ defined the interval-valued intuitionistic fuzzy weighted arithmetic (IIFWA) operator, the interval-valued intuitionistic fuzzy ordered weighted geometric (IIFOWG) operator and the interval-valued intuitionistic fuzzy hybrid geometric (IIFHG) operator. Xu and Chen^27^ defined the interval-valued intuitionistic fuzzy ordered weighted averaging (IIFOWA) operator, and Xu^11^ proposed some operators of IVIFVs and the corresponding ranking methods. Kong et al.^28^ utilized the IIFWA operator to aggregate the utility evaluation matrix and the preference information of decision-makers and established the combinatorial optimization model of the interval-valued intuitionistic fuzzy MAGDM problem based on the combinatorial optimization method of decision variables.

The above methods are effective in solving some decision-making problems in interval-valued intuitionistic fuzzy environments. However, they still have the following drawbacks:

1. Utilizing the IIFWA^26^ operator to aggregate the information given by DMs, if the nonmembership degree of an IVIFV is zero, then the nonmembership degree of the IVIFVs aggregated by the operator must be zero, even if the nonmembership degrees of other IVIFVs are not zero. Therefore, the nonmembership degree of other IVIFVs cannot be reflected in the decision-making process. In particular, the IIFWA operator reflects the role of the group, ignores the role of individuals in decision-making and is insensitive to the role of individuals. Thus, the aggregated information will result in the loss or distortion of the original decision information in the aggregation process and lead to decision error. Therefore, the application of the IIFWA operator in decision-making will introduce decision error.
2. Researchers have developed many useful methods to rank the order of alternatives. In some studies^18–22^, the order of alternatives is ranked based on methods including distance functions, score functions, correlation coefficients and the TOPSIS method. However, the methods do not make sufficient use of the IVIFV information, which may cause the loss of information and will inevitably affect the ranking validity and accuracy. The identification degree of the final evaluation results of an alternative is different when using different decision-making methods. When the attribute values of different alternatives are very close to each other, the final evaluation results of alternatives may be very close to one another or even equal; thus, it is difficult to distinguish alternatives. In this situation, the effectiveness of the ranking order of alternatives remains to be discussed.
3. In some studies^25–28^, the MAGDM method based on aggregation operators was proposed. These methods provide the DMs’ weight and attributes’ weight as crisp numbers, which makes it difficult to avoid randomness, and it is assumed that the weight of the same attribute given by different DMs is the same. Thus, it is difficult to reflect the different preferences of DMs. Since decision-makers usually come from various research areas and there may be many differences in their knowledge structure, expression abilities, evaluation levels, preferences and practical experience, the corresponding opinions or evaluations of DMs may differ substantially. Therefore, different DMs usually weight the same attribute differently. Thus, these methods are inappropriate for MAGDM problems with variable attributes. Therefore, how to solve these restrictions is the focus of our work. Yao^29^ studied an MAGDM problem where the weights of the attributes given by decision-makers are different, and proposed a MAGDM method based on the preference order and the improved score function

To overcome the above-stated limitations, we investigate group decision-making problems in interval-valued intuitionistic fuzzy environments. The innovations and novelties of this paper are summarized as follows:

1. Some operational laws of IVIFVs are redefined, and a new IVIFV aggregation operator is defined to overcome the drawbacks of the existing operations and IIFWA aggregation operator of IVIFVs. The new aggregation operator takes the role of the nonmembership degree of each IVIFV into account in decision-making, reduces the loss or distortion of the original decision information in the process of aggregation and improves the sensitivity to individuals.
2. The means and variances of IVIFVs based on the concept of definite integrals are defined to provide more comprehensive decision-making information for the decision-making process.
3. Combining the means and variances of alternatives on attributes, the adjusted comprehensive values for attributes are formed. Thus the evaluation results can better reflect the difference between different alternatives, improve the identification of the evaluation results and make it easier to sort and select the best alternatives.
4. Based on the means and variances of the IVIFVs, a novel MAGDM method, in which the weights of the attributes are different for different DMs, is proposed to better address uncertainty in the MAGDM problem.

The remainder of this paper is organized as follows. In "Preliminaries", we present some definitions of IVIFVs, the IIFWA aggregation operator, score function, possibility degree and ordered formula of IVIFVs. In "Analysis of the drawbacks of IIFWA operators and the proposal of an improved IVIFV operator", we analyse the shortcomings of the IIFWA operator and propose an improved interval-valued intuitionistic fuzzy weighted arithmetic averaging (IIIFWAA) aggregation operator to overcome the drawbacks of the IIFWA operator. In "The means and variances of the possibility degrees of IVIFVs", based on the possibility degree of IVIFVs, we propose the means and variances of the IVIFVs to overcome the second limitation. In "A new method for MAGDM with IVIFVs", we propose a new MAGDM method based on the proposed IIIFWAA operator and the means and variances of the IVIFVs. The proposed method is applied to an air combat training accuracy assessment example, and the results are comparatively analysed and discussed with other existing methods. Finally, conclusions , limitations and possible future work are given in "Conclusions, limitations, and future works".

## Preliminaries

This section introduces some basic concepts and definitions for the IVIFVs, scores function and possibility degree function.

### Interval-valued intuitionistic fuzzy sets

#### Definition 1

Let Z be a nonempty finite universe. An interval-valued intuitionistic fuzzy set $\tilde{A}$ in Z is defined as $\tilde{A} = \left\{ {\;(\;z,\;[\mu_{{\tilde{A}}}^{ - } (z)\;,\mu_{{\tilde{A}}}^{ + } (z)],\;[\nu_{{\tilde{A}}}^{ - } (z)\;,\nu_{{\tilde{A}}}^{ + } (z)])\left| {z \in Z} \right|} \right\}$, where $[\mu_{{\tilde{A}}}^{ - } (z)\;,\mu_{{\tilde{A}}}^{ + } (z)] \subseteq [0,1]$ and $\;[\nu_{{\tilde{A}}}^{ - } (z)\;,\nu_{{\tilde{A}}}^{ + } (z)]) \subseteq [0,1][0,1]$ with the condition $0 \le \mu_{{\tilde{A}}}^{ + } (z) + \nu_{{\tilde{A}}}^{ + } (z) \le 1$ for all $z \in Z$. The intervals $[\mu_{{\tilde{A}}}^{ - } (z)\;,\mu_{{\tilde{A}}}^{ + } (z)]$ and $[\mu_{{\tilde{A}}}^{ - } (z)\;,\mu_{{\tilde{A}}}^{ + } (z)]$ represent the interval of the membership degree and the interval of the nonmembership degree of element z to IVIFS $\tilde{A}$, respectively^16^.

#### Definition 2

Assume that $\tilde{\alpha }_{1} = \left( {\left[ {\mu_{1}^{ - } ,\mu_{1}^{ + } } \right],\left[ {\nu_{1}^{ - } ,\nu_{1}^{ + } } \right]} \right)$ and $\tilde{\alpha }_{2} = \left( {\left[ {\mu_{2}^{ - } ,\mu_{2}^{ + } } \right],\left[ {\nu_{2}^{ - } ,\nu_{2}^{ + } } \right]} \right)$ are any two IVIFVs^30^. Then, the operational laws of IVIFVs are defined as

1 $$\tilde{\alpha }_{1} \oplus \tilde{\alpha }_{2} = \left( {\left[ {\mu_{1}^{ - } + \mu_{2}^{ - } - \mu_{1}^{ - } \mu_{2}^{ - } ,\mu_{1}^{ + } + \mu_{2}^{ + } - \mu_{1}^{ + } \mu_{2}^{ + } } \right],\left[ {\nu_{1}^{ - } \nu_{2}^{ - } ,\nu_{1}^{ + } \nu_{2}^{ + } } \right]} \right)$$

2 $$\lambda \cdot \tilde{\alpha }_{1}^{{}} = \left( {\left[ {1 - (1 - \mu_{1}^{ - } )^{\lambda } ,1 - (1 - \mu_{1}^{ + } )^{\lambda } } \right],\left[ {\left( {\nu_{1}^{ - } } \right)^{\lambda } ,\left( {\nu_{1}^{ + } } \right)^{\lambda } } \right]} \right),\lambda \ge 0$$

#### Definition 3

Let $\tilde{\alpha }_{j} = \left( {\left[ {\mu_{j}^{ - } ,\mu_{j}^{ + } } \right],\left[ {\nu_{j}^{ - } ,\nu_{j}^{ + } } \right]} \right)\left( {j = 1,2, \cdots ,n} \right)$ be a collection of IVIFVs, and $\omega = \left( {\omega_{1} ,\omega_{2} , \cdots ,\omega_{n} } \right)^{T}$ be their associated weight vector, where $\omega_{j} \in \left[ {0,1} \right]$ and $\sum\nolimits_{j = 1}^{n} {\omega_{j} = 1}$^30^, then the interval-valued intuitionistic fuzzy weighted arithmetic (IIFWA) aggregation operator is defined as:

3 $$IIFWA(\tilde{\alpha }_{1} ,\tilde{\alpha }_{2} , \cdots \tilde{\alpha }_{n} ) = \left( {\left[ {1 - \prod\limits_{j = 1}^{n} {(1 - \mu_{j}^{ - } )^{{\omega_{j} }} } ,1 - \prod\limits_{j = 1}^{n} {(1 - \mu_{j}^{ + } )^{{\omega_{j} }} } } \right],\;\;\left[ {\prod\limits_{j = 1}^{n} {(\nu_{j}^{ - } )^{{\omega_{j} }} } \;,\prod\limits_{j = 1}^{n} {(\nu_{j}^{ + } )^{{\omega_{j} }} } } \right]} \right).$$

### The score function and possibility degree

#### Definition 4

Let $\tilde{\alpha } = \left( {\left[ {\mu_{{}}^{ - } ,\mu_{{}}^{ + } } \right],\left[ {\nu_{{}}^{ - } ,\nu_{{}}^{ + } } \right]} \right)$ be an IVIFV. Xu’s score function $S\left( {\tilde{\alpha }} \right)$^30^ of $\tilde{\alpha }$ is defined as follows:

4 $$S(\tilde{\alpha }) = \frac{{\mu^{ - } + \mu^{ + } - \nu^{ - } - \nu^{ + } }}{2}.$$

#### Definition 5

For the intervals $\alpha = [\alpha^{ - } ,\alpha^{ + } ]$ and $\beta = [\beta^{ - } ,\beta^{ + } ]$, the possibility degree of $\alpha \ge \beta$^31^ is defined as follows:

5 $$p(\alpha \ge \beta ) = \frac{{\max (0,\alpha^{ + } - \alpha^{ - } + \beta^{ + } - \beta^{ - } - \max (0,\beta^{ + } - \alpha^{ - } ))}}{{\alpha^{ + } + \beta^{ + } - \alpha^{ - } - \beta^{ - } }}$$

#### Definition 6

Assume that $P = (p_{ij} )_{m \times m}$ is a complement judgement matrix; then its ordered vector^26^ $R = (r(p_{1} ),r(p_{2} ), \cdots r(p_{m} ))^{T}$ is defined as follows:

6 $$r(p_{i} ) = \frac{{\sum\nolimits_{j = 1}^{m} {p_{ij} + \frac{m - 2}{2}} }}{m(m - 1)},\;\;\;\;{\kern 1pt} i = 1,2, \cdots m.$$

## Analysis of the drawbacks of IIFWA operators and the proposal of an improved IVIFV operator

In this section, we analyse the drawbacks of the IIFWA aggregation operator in some situations. To eliminate the drawbacks, the novel operational laws of IVIFVs and the improved interval-valued intuitionistic fuzzy weighted arithmetic averaging (IIIFWAA) aggregation operator are defined.

### Example 3.1

For one decision-making problem, let A1 and A2 be two alternatives, let C1 and C2 be two benefit type attributes and let w1 and w2 be the weights of the attributes C1 and C2 given by the decision-maker, respectively, where w1 = w2 = 0.5. Assume the decision-maker evaluates the alternatives with respect to the attributes by using IVIFVs to provide the decision matrix, which is shown as follows:

$$B = (\tilde{b}_{ij} )_{2 \times 2} = \begin{array}{*{20}c} {} \\ {A1} \\ {A2} \\ \end{array} \begin{array}{*{20}c} {\begin{array}{*{20}c} {C1} & {\quad \quad \quad \quad \quad \quad } \\ \end{array} C2} \\ {\left( {\begin{array}{*{20}c} {([0.6,0.6],[0,0])} & {([0.8,0.8],[0.2,0.2])} \\ {([0.5,0.5],[0,0])} & {([0.84,0.84],[0.16,0.16])} \\ \end{array} } \right)} \\ \end{array}$$

Using the IIFWA aggregation operator shown in Eq. (3), we obtain $IIFWA({\tilde{\text{b}}}_{11} ,\tilde{b}_{12} {) = }\left( {{[0}{\text{.717,0}}{.717],[0,0]}} \right)$ and $IIFWA({\tilde{\text{b}}}_{21} ,\tilde{b}_{22} {) = }\left( {{[0}{\text{.717,0}}{.717],[0,0]}} \right)$. Because the nonmembership degree of $\tilde{b}_{11}$ and $\tilde{b}_{21}$ is 0, it is obvious that the non-membership degrees of the IVIFVs integrated by the IIFWA aggregation operators are all zero. Therefore, the role of the nonmembership degree of IVIFVs $\tilde{b}_{12}$ and $\tilde{b}_{22}$ cannot be reflected in the decision-making process. Because the IVIFVs $\tilde{b}_{11}$ and $\tilde{b}_{12}$ appear in the first row of decision matrix B, the IVIFVs $\tilde{b}_{21}$ and $\tilde{b}_{22}$ that appear in the second row of decision matrix B are different; therefore, the aggregation of attributes for alternatives A1 and A2 should be different. However, according to the IIFWA aggregation operator shown in Eq. (3), it can be seen that $IIFWA({\tilde{\text{b}}}_{11} ,\tilde{b}_{12} {) = }IIFWA({\tilde{\text{b}}}_{21} ,\tilde{b}_{22} )$. Therefore, the drawback of the IIFWA aggregation operators is that they cannot distinguish the preference order of alternatives A1 and A2 in this case.

### Example 3.2

For one decision-making problem, let A1 and A2 be two alternatives, let C1 and C2 be two benefit type attributes and let w1 and w2 be the weights of the attributes C1 and C2 given by the decision-maker, respectively, where w1 = w2 = 0.5. Assume the decision-maker evaluates the alternatives with respect to the attributes by using IVIFVs to provide the decision matrix, which is shown as follows:

$$D = (\tilde{d}_{ij} )_{2 \times 2} = \begin{array}{*{20}c} {} \\ {A1} \\ {A2} \\ \end{array} \begin{array}{*{20}c} {\begin{array}{*{20}c} {C1} & {\quad \quad \quad \quad \quad \quad } \\ \end{array} C2} \\ {\left( {\begin{array}{*{20}c} {([0.4,0.4],[0,0.5])} & {([0.2,0.2],[0.1,0.5])} \\ {([0.25,0.25],[0.45,0.5])} & {([0.36,0.36],[0,0.5])} \\ \end{array} } \right)} \\ \end{array} .$$

Using the IIFWA aggregation operator shown in Eq. (3), $IIFWA(\tilde{d}_{11} ,\tilde{d}_{12} ) = \left( {[0.3072,0.3072],[0,0.5]} \right)$ and $IIFWA(\tilde{d}_{21} ,\tilde{d}_{22} ) = \left( {[0.3072,0.3072],[0,0.5]} \right)$. Because the lower bound of the nonmembership degrees of $\tilde{d}_{11}$ and $\tilde{d}_{22}$ are both zero, it is obvious that the lower bounds of the nonmembership degrees of IVIFVs integrated by the IIFWA aggregation operator are all zero. Thus, the role of the nonmembership degree of IVIFVs $\tilde{d}_{12}$ and $\tilde{d}_{21}$ cannot be reflected in the decision-making process. Because the IVIFVs $\tilde{d}_{11}$ and $\tilde{d}_{12}$ appear in the first row of decision matrix D, the IVIFVs $\tilde{d}_{21}$ and $\tilde{d}_{22}$ that appear in the second row of decision matrix D are different; therefore, the aggregation of attributes for alternatives A1 and A2 should be different. However, according to the IIFWA aggregation operator shown in Eq. (3), it is observed that $IIFWA(\tilde{d}_{11} ,\tilde{d}_{12} ) = IIFWA(\tilde{d}_{21} ,\tilde{d}_{22} )$. Therefore, the drawback of IIFWA aggregation operators is that they cannot distinguish the preference order of alternatives A1 and A2 in this case.

Based on the above-stated analysis, we can see that the IIFWA aggregation operator in Definition 3, which is defined by using the operations of IVIFVs in Definition 2, overemphasizes the role of groups and is not sensitive to individuals. When the non-membership degree of an attribute is zero, the non-membership degree of the IVIFV integrated by the IIFWA operator must be zero. Thus, the IIFWA aggregation operator will lose or distort the original decision information in the process of aggregation. This will cause decision errors in the multiattribute group decision-making process. Thus, the improved operations and integrating operator of IVIFVs are defined as follows.

### Definition 7

Assume that $\tilde{\alpha }_{1} = \left( {\left[ {\mu_{1}^{ - } ,\mu_{1}^{ + } } \right],\left[ {\nu_{1}^{ - } ,\nu_{1}^{ + } } \right]} \right)$ and $\tilde{\alpha }_{2} = \left( {\left[ {\mu_{2}^{ - } ,\mu_{2}^{ + } } \right],\left[ {\nu_{2}^{ - } ,\nu_{2}^{ + } } \right]} \right)$ are any two IVIFVs, and then the novel operational laws of IVIFVs are defined as follows:

7 $$\tilde{\alpha }_{1} \oplus \tilde{\alpha }_{2} = \left( \begin{gathered} \left[ {1 - \left( {1 - \mu_{{1}}^{ - } } \right)\left( {1 - \mu_{{2}}^{ - } } \right),\;\;1 - \left( {1 - \mu_{{1}}^{ + } } \right)\left( {1 - \mu_{{2}}^{ + } } \right)} \right] \hfill \\ \left[ {\left( {1 - \mu_{{1}}^{ - } } \right)\left( {1 - \mu_{{2}}^{ - } } \right) - \left( {1 - \mu_{{1}}^{ - } - \nu_{1}^{ - } } \right)\left( {1 - \mu_{{2}}^{ - } - \nu_{2}^{ - } } \right),\;\;\left( {1 - \mu_{{1}}^{ + } } \right)\left( {1 - \mu_{{2}}^{ + } } \right) - \left( {1 - \mu_{{1}}^{ + } - \nu_{1}^{ + } } \right)\left( {1 - \mu_{{2}}^{ + } - \nu_{2}^{ + } } \right)} \right] \hfill \\ \end{gathered} \right),$$

8 $$\lambda \tilde{\alpha }_{1} = \left( {\left[ {1 - \left( {1 - \mu_{{1}}^{ - } } \right)^{\lambda } ,\;\;1 - \left( {1 - \mu_{{1}}^{ + } } \right)^{\lambda } } \right],\left[ {\left( {1 - \mu_{{1}}^{ - } } \right)^{\lambda } - \left( {1 - \mu_{{1}}^{ - } - \nu_{1}^{ - } } \right)^{\lambda } ,\;\;\left( {1 - \mu_{{1}}^{ + } } \right)^{\lambda } - \left( {1 - \mu_{{1}}^{ + } - \nu_{1}^{ + } } \right)^{\lambda } } \right]} \right).$$

The results calculated by using Eqs. (7) and (8) are still IVIFVs. The improved operations have the following properties:

1. $$\tilde{\alpha }_{1} \oplus \tilde{\alpha }_{2} = \tilde{\alpha }_{2} \oplus \tilde{\alpha }_{1}$$;
2. $$(\tilde{\alpha }_{1} \oplus \tilde{\alpha }_{2} ) \oplus \tilde{\alpha }_{3} = \tilde{\alpha }_{1} \oplus (\tilde{\alpha }_{2} \oplus \tilde{\alpha }_{3} )$$;
3. $$\lambda (\tilde{\alpha }_{1} \oplus \tilde{\alpha }_{2} ) = \lambda \tilde{\alpha }_{1} \oplus \lambda \tilde{\alpha }_{2}$$.

Based on the operational laws of IVIFVs in Definition 7, the improved interval-valued intuitionistic fuzzy weighted arithmetic averaging (IIIFWAA) aggregation operator is defined as follows:

### Definition 8

Assume that $\tilde{\alpha }_{j} = \left( {\left[ {\mu_{j}^{ - } ,\mu_{j}^{ + } } \right],\left[ {\nu_{j}^{ - } ,\nu_{j}^{ + } } \right]} \right)\left( {j = 1,2, \cdots ,n} \right)$ is a collection of IVIFVs, and then the improved interval-valued intuitionistic fuzzy weighted arithmetic averaging (IIIFWAA) aggregation operator is defined as follows:

9 $$IIIFWAA(\tilde{\alpha }_{1} ,\tilde{\alpha }_{2} , \cdots \tilde{\alpha }_{n} ) = \left( \begin{gathered} \;\;\left[ {1 - \prod\limits_{j = 1}^{n} {(1 - \mu_{j}^{ - } )^{{\omega_{j} }} } ,1 - \prod\limits_{j = 1}^{n} {(1 - \mu_{j}^{ + } )^{{\omega_{j} }} } } \right]\;,\;\; \hfill \\ \left[ {\prod\limits_{j = 1}^{n} {(1 - \mu_{j}^{ - } )^{{\omega_{j} }} } - \prod\limits_{j = 1}^{n} {(1{ - }\mu_{j}^{ - } { - }\nu_{j}^{ - } )^{{\omega_{j} }} } \;,\prod\limits_{j = 1}^{n} {(1 - \mu_{j}^{ + } )^{{\omega_{j} }} } - \prod\limits_{j = 1}^{n} {(1{ - }\mu_{j}^{ + } { - }\nu_{j}^{ + } )^{{\omega_{j} }} } \;} \right] \hfill \\ \end{gathered} \right),$$

where $\omega = \left( {\omega_{1} ,\omega_{2} , \cdots ,\omega_{n} } \right)^{T}$ is the weight vector of $\tilde{\alpha }_{j} \left( {j = 1,2, \cdots ,n} \right)$, where $\omega_{j} \in \left[ {0,1} \right]$ and $\sum\nolimits_{j = 1}^{n} {\omega_{j} = 1}$.

### Proof.

When n = 1, Eq. (9) degenerates into Eq. (8).

1. When n = 2, according to Eq. (7) and Eq. (8), we can obtain
  $$\begin{gathered} \omega_{1} \tilde{\alpha }_{1} = \left( {\left[ {1 - \left( {1 - \mu_{1}^{ - } } \right)^{{\omega_{1} }} ,1 - \left( {1 - \mu_{1}^{ + } } \right)^{{\omega_{1} }} } \right],\left[ {\left( {1 - \mu_{1}^{ - } } \right)^{{\omega_{1} }} - \left( {1 - \mu_{1}^{ - } - \nu_{1}^{ - } } \right)^{{\omega_{1} }} ,\left( {1 - \mu_{1}^{ + } } \right)^{{\omega_{1} }} - \left( {1 - \mu_{1}^{ + } - \nu_{1}^{ + } } \right)^{{\omega_{1} }} } \right]} \right) \hfill \\ \omega_{2} \tilde{\alpha }_{2} = \left( {\left[ {1 - \left( {1 - \mu_{2}^{ - } } \right)^{{\omega_{2} }} ,1 - \left( {1 - \mu_{2}^{ + } } \right)^{{\omega_{2} }} } \right],\left[ {\left( {1 - \mu_{2}^{ - } } \right)^{{\omega_{2} }} - \left( {1 - \mu_{2}^{ - } - \nu_{2}^{ - } } \right)^{{\omega_{2} }} ,\left( {1 - \mu_{2}^{ + } } \right)^{{\omega_{2} }} - \left( {1 - \mu_{2}^{ + } - \nu_{2}^{ + } } \right)^{{\omega_{2} }} } \right]} \right) \hfill \\ \omega_{1} \tilde{\alpha }_{1} + \omega_{2} \tilde{\alpha }_{2} = \left( \begin{gathered} \left[ {1 - \left( {1 - \mu_{1}^{ - } } \right)^{{\omega_{1} }} \left( {1 - \mu_{2}^{ - } } \right)^{{\omega_{2} }} ,1 - \left( {1 - \mu_{1}^{ + } } \right)^{{\omega_{1} }} \left( {1 - \mu_{2}^{ + } } \right)^{{\omega_{2} }} } \right], \hfill \\ \left[ \begin{gathered} \left( {1 - \mu_{1}^{ - } } \right)^{{\omega_{1} }} \left( {1 - \mu_{2}^{ - } } \right)^{{\omega_{2} }} - \left( {1 - \mu_{1}^{ - } - \nu_{1}^{ - } } \right)^{{\omega_{1} }} \left( {1 - \mu_{2}^{ - } - \nu_{2}^{ - } } \right)^{{\omega_{2} }} , \hfill \\ \left( {1 - \mu_{1}^{ + } } \right)^{{\omega_{1} }} \left( {1 - \mu_{2}^{ + } } \right)^{{\omega_{2} }} - \left( {1 - \mu_{1}^{ + } - \nu_{1}^{ + } } \right)^{{\omega_{1} }} \left( {1 - \mu_{2}^{ + } - \nu_{2}^{ + } } \right)^{{\omega_{2} }} \hfill \\ \end{gathered} \right] \hfill \\ \end{gathered} \right) \hfill \\ \end{gathered}$$
2. Suppose that n = k. Then, Eq. (9) holds, and
  $$IIIFWAA\left( {\tilde{\alpha }_{1} ,\tilde{\alpha }_{2} , \cdots ,\tilde{\alpha }_{k} } \right) = \left( \begin{gathered} \left[ {1 - \prod\limits_{j = 1}^{k} {\left( {1 - \mu_{j}^{ - } } \right)^{{\omega_{j} }} ,} 1 - \prod\limits_{j = 1}^{k} {\left( {1 - \mu_{j}^{ + } } \right)^{{\omega_{j} }} } } \right], \hfill \\ \left[ {\prod\limits_{j = 1}^{k} {\left( {1 - \mu_{j}^{ - } } \right)^{{\omega_{j} }} - \prod\limits_{j = 1}^{k} {\left( {1 - \mu_{j}^{ - } - \nu_{j}^{ - } } \right)^{{\omega_{j} }} ,\prod\limits_{j = 1}^{k} {\left( {1 - \mu_{j}^{ + } } \right)^{{\omega_{j} }} - \prod\limits_{j = 1}^{k} {\left( {1 - \mu_{j}^{ + } - \nu_{j}^{ + } } \right)^{{\omega_{j} }} ,} } } } } \right] \hfill \\ \end{gathered} \right)$$
3. When n = k + 1, we obtain
  $$\begin{aligned} IIIFWAA(\tilde{\alpha }_{1} ,\alpha_{2} , \cdots ,\tilde{\alpha }_{k + 1} ) & = \left( \begin{gathered} \left[ {1 - \prod\limits_{j = 1}^{k} {\left( {1 - \mu_{j}^{ - } } \right)^{{\omega_{j} }} ,} 1 - \prod\limits_{j = 1}^{k} {\left( {1 - \mu_{j}^{ + } } \right)^{{\omega_{j} }} } } \right], \hfill \\ \, \left[ {\prod\limits_{j = 1}^{k} {\left( {1 - \mu_{j}^{ - } } \right)^{{\omega_{j} }} } - \prod\limits_{j = 1}^{k} {\left( {1 - \mu_{j}^{ - } - \nu_{j}^{ - } } \right)^{{\omega_{j} }} ,} \prod\limits_{j = 1}^{k} {\left( {1 - \mu_{j}^{ + } } \right)^{{\omega_{j} }} - \prod\limits_{j = 1}^{k} {\left( {1 - \mu_{j}^{ + } - \nu_{j}^{ + } } \right)^{{\omega_{j} }} } } } \right] \hfill \\ \end{gathered} \right) \\ & \quad \oplus \left( \begin{gathered} \left[ {1 - \left( {1 - \mu_{k + 1}^{ - } } \right)^{{\omega_{k + 1} }} ,1 - \left( {1 - \mu_{k + 1}^{ + } } \right)^{{\omega_{k + 1} }} } \right], \hfill \\ \left[ {\left( {1 - \mu_{k + 1}^{ - } } \right)^{{\omega_{k + 1} }} - \left( {1 - \mu_{k + 1}^{ - } - \nu_{k + 1}^{ - } } \right)^{{\omega_{k + 1} }} ,\left( {1 - \mu_{k + 1}^{ + } } \right)^{{\omega_{k + 1} }} - \left( {1 - \mu_{k + 1}^{ + } - \nu_{k + 1}^{ + } } \right)^{{\omega_{k + 1} }} } \right] \hfill \\ \end{gathered} \right) \\ & = \left( \begin{gathered} \left[ \begin{gathered} 1 - \left( {1 - \left( {1 - \prod\limits_{j = 1}^{k} {\left( {1 - \mu_{j}^{ - } } \right)^{{\omega_{j} }} } } \right)} \right)\left( {1 - \left( {1 - \left( {1 - \mu_{k + 1}^{ - } } \right)^{{\omega_{k + 1} }} } \right)} \right), \hfill \\ 1 - \left( {1 - \left( {1 - \prod\limits_{j = 1}^{k} {\left( {1 - \mu_{j}^{ + } } \right)^{{\omega_{j} }} } } \right)} \right)\left( {1 - \left( {1 - \left( {1 - \mu_{k + 1}^{ + } } \right)^{{\omega_{k + 1} }} } \right)} \right) \hfill \\ \end{gathered} \right] \hfill \\ \left[ \begin{gathered} \prod\limits_{j = 1}^{k + 1} {\left( {1 - \mu_{j}^{ - } } \right)^{{\omega_{j} }} } \cdot \left( {1 - \mu_{k + 1}^{ - } } \right)^{{\omega_{k + 1} }} - \left( {\prod\limits_{j = 1}^{k + 1} {\left( {1 - \mu_{j}^{ - } } \right)^{{\omega_{j} }} } - \left( {\prod\limits_{j = 1}^{k} {\left( {1 - \mu_{j}^{ - } } \right)^{{\omega_{j} }} } - \prod\limits_{j = 1}^{k} {\left( {1 - \mu_{j}^{ - } - \nu_{j}^{ - } } \right)^{{\omega_{j} }} } } \right)} \right)\left( {1 - \mu_{k + 1}^{ - } - \nu_{k + 1}^{ - } } \right)^{{\omega_{k + 1} }} , \hfill \\ \prod\limits_{j = 1}^{k + 1} {\left( {1 - \mu_{j}^{ + } } \right)^{{\omega_{j} }} } \cdot \left( {1 - \mu_{k + 1}^{ + } } \right)^{{\omega_{k + 1} }} - \left( {\prod\limits_{j = 1}^{k + 1} {\left( {1 - \mu_{j}^{ + } } \right)^{{\omega_{j} }} } - \left( {\prod\limits_{j = 1}^{k} {\left( {1 - \mu_{j}^{ + } } \right)^{{\omega_{j} }} } - \prod\limits_{j = 1}^{k} {\left( {1 - \mu_{j}^{ + } - \nu_{j}^{ + } } \right)^{{\omega_{j} }} } } \right)} \right)\left( {1 - \mu_{k + 1}^{ + } - \nu_{k + 1}^{ + } } \right)^{{\omega_{k + 1} }} \hfill \\ \end{gathered} \right] \hfill \\ \end{gathered} \right) \\ & = \left( \begin{gathered} \left[ {1 - \prod\limits_{j = 1}^{k + 1} {\left( {1 - \mu_{j}^{ - } } \right)^{{\omega_{j} }} } ,1 - \prod\limits_{j = 1}^{k + 1} {\left( {1 - \mu_{j}^{ + } } \right)^{{\omega_{j} }} } } \right], \hfill \\ \left[ {\prod\limits_{j = 1}^{k + 1} {\left( {1 - \mu_{j}^{ - } } \right)^{{\omega_{j} }} } - \prod\limits_{j = 1}^{k + 1} {\left( {1 - \mu_{j}^{ - } - \nu_{j}^{ - } } \right)^{{\omega_{j} }} ,} \prod\limits_{j = 1}^{k + 1} {\left( {1 - \mu_{j}^{ + } } \right)^{{\omega_{j} }} } - \prod\limits_{j = 1}^{k + 1} {\left( {1 - \mu_{j}^{ + } - \nu_{j}^{ + } } \right)^{{\omega_{j} }} } } \right] \hfill \\ \end{gathered} \right) \\ \end{aligned}$$

Therefore, Eq. (9) holds when n = k + 1. By combining (1), (2) and (3), it can be seen that Eq. (9) is true for all n.

In the following, we will demonstrate that the proposed operator (IIIFWAA) can be used to address the drawbacks of the IIFWA operator shown in Examples 3.1 and 3.2.

### Example 3.3

Assume the same data as that in Example 3.1. Based on the proposed new aggregation operator shown in Eq. (9), we obtain $IIIFWAA_{A1} (\tilde{b}_{11} ,\tilde{b}_{12} ) = \left( {[0.717,0.717],[0.0828,0.0828]} \right)$ and $IIIFWAA_{A2} (\tilde{b}_{21} ,\tilde{b}_{22} ) = \left( {[0.717,0.717],[0.0483,0.0483]} \right)$. According to Xu’s score function $S(\tilde{\alpha })$ shown in Eq. (4), S(A1) = 0.6342 and S(A2) = 0.6687. Because S(A1) < S(A2), the ranking order between alternatives A1 and A2 is A1 < A 2. Thus, the proposed IIIFWAA aggregation operator can be used to address the shortcoming of the IIFWA operator, which cannot distinguish the PO of alternatives A1 and A2, as shown in Example 3.1.

### Example 3.4

Assume the same data as that in Example 3.2. Based on the proposed aggregation operator shown in Eq. (9), we obtain $IIIFWAA_{A1} (\tilde{d}_{11} ,\tilde{d}_{12} ) = \left( {[0.3072,0.3072],[0.0447,0.5196]} \right)$ and $IIIFWAA_{A1} (\tilde{d}_{21} ,\tilde{d}_{22} ) = \left( {[0.3072,0.3072],[0.2546,0.5057]} \right)$. According to Xu’s score function $S(\tilde{\alpha })$ shown in Eq. (4), $S(A1) = 0.02505$ and $S(A2) = - 0.07295$. Because S(A1) > S(A2), the ranking order between alternatives A1 and A2 is A1 > A2. Thus, the proposed IIIFWAA aggregation operator can be used to address the shortcoming of the IIFWA operator (Xu 2007), which cannot distinguish the PO of alternatives A1 and A2, as shown in Example 3.2.

Based on the above discussions, we can see that the non-membership degrees of the IVIFVs integrated by the improved operator are not zero. Since the improved IIIFWAA aggregation operator takes the role of the membership degree and non-membership degree of each attribute into account in the decision-making process, it emphasizes the role of individuals and is sensitive to individuals. Therefore, we can see that the new IIIFWAA aggregation operator shown in Eq. (9) can overcome the shortcomings of Xu’s IIFWA operator.

## The means and variances of the possibility degrees of IVIFVs

In multiattribute decision-making problems, the attribute values of some alternatives are usually very close to those of other alternatives. This can lead to the comprehensive values of some alternatives being the same or very close by using some decision-making methods. Therefore, it is difficult to distinguish these alternatives. Because the identification degree of the alternatives is not obvious, it is difficult to ensure the validity and accuracy of the ranking order. In the following, we use one example to demonstrate this situation.

### Example 4.1

Let A1, A2, A3 and A4 be four alternatives, let C1, C2 and C3 be three benefit type attributes and let w1, w2 and w3 be the weights of the attributes C1, C2 and C3 given by the decision-maker, respectively, where w1 = 0.2, w2 = 0.3 and w3 = 0.5. Assume the decision-maker evaluates the alternatives with respect to the attributes by using IVIFVs to provide the decision matrix, which is shown as follows:

$$M = (\tilde{a}_{ij} )_{4 \times 3} = \begin{array}{*{20}c} {A1} \\ {A2} \\ \begin{gathered} A3 \hfill \\ A4 \hfill \\ \end{gathered} \\ \end{array} \begin{array}{*{20}c} {\begin{array}{*{20}c} {C1} & {\quad \quad \quad \quad \quad \quad \quad \quad \quad C2\quad \quad \quad \quad \quad \quad \quad \quad } \\ \end{array} C3} \\ {\left( {\begin{array}{*{20}c} {([0.5,0.6],[0.2,0.3])} & {([0.61,0.7],[0.2,0.3])} & {([0.5,0.6],[0.2,0.3])} \\ {([0.51,0.61],[0.22,0.31])} & {([0.6,0.6],[0.21,0.3])} & {([0.49,0.58],[0.2,0.3])} \\ {([0.52,0.62],[0.1,0.33])} & {([0.58,0.61],[0.2,0.3])} & {([0.55,0.58],[0.2,0.4])} \\ {([0.54,0.6],[0.2,0.22])} & {([0.62,0.59],[0.2,0.4])} & {([0.52,0.59],[0.19,0.3])} \\ \end{array} } \right)} \\ \end{array} .$$

The evaluations of the alternatives are aggregated with weights by using the IIIFWAA aggregation operators in Eq. (9). The aggregated IVIFVs are shown as follows:

$$IIIFWAA_{A1} = ([0.5359,0.6331],[0.2025,0.3669]),IIIFWAA_{A2} = \left( {\left[ {0.5296, \, 0.5922} \right],\left[ {0.2100,0.3030} \right]} \right),$$

$$IIIFWAA_{A3} = \left( {\left[ {0.5535,0.5974} \right],\left[ {0.1849,0.3649} \right]} \right),IIIFWAA_{A4} = \left( {\left[ {0.5563,0.5920} \right],\left[ {0.1978,0.3489} \right]} \right).$$

According to Eq. (4), S(A1) = 0.2998, S(A2) = 0.3044, S(A3) = 0.3005, and S(A4) = 0.3008. The alternatives are sorted according to the score value, and the ordered result is A2 > A4 > A3 > A1. From the score values, we can see that the scores are very close, and the difference between A3 and A4 is only 0.0003.

From the comparison of the score values of alternatives, the difference between the score values is very small. Therefore, the identification degree of the alternatives is not obvious. It is difficult to ensure the validity and accuracy of the ranking order. Thus, it is desirable to develop a new method that can both reflect the difference between the evaluation results and ensure the effectiveness and accuracy of the decision-making result. In this paper, the mean and variance of the IVIFVs are defined based on the possibility degrees and the idea of a definite integral.

### Definition 9

Let $\tilde{\alpha }_{1} = \left( {\left[ {\mu_{1}^{ - } ,\mu_{1}^{ + } } \right],\left[ {\nu_{1}^{ - } ,\nu_{1}^{ + } } \right]} \right)$ and $\tilde{\alpha }_{2} = \left( {\left[ {\mu_{2}^{ - } ,\mu_{2}^{ + } } \right],\left[ {\nu_{2}^{ - } ,\nu_{2}^{ + } } \right]} \right)$ be any two IVIFVs, the variable $x \in [0,1]$. Let $\alpha_{1}^{ - } (x) = \mu_{1}^{ - } + x \cdot (1 - \mu_{1}^{ - } - \nu_{1}^{ + } ), \, \alpha_{1}^{ + } (x) = \mu_{1}^{ + } + x \cdot (1 - \mu_{1}^{ + } - \nu_{1}^{ - } )$, $\alpha_{2}^{ - } (x) = \mu_{2}^{ - } + x \cdot (1 - \mu_{2}^{ - } - \nu_{2}^{ + } )$, and $\alpha_{2}^{ + } (x) = \mu_{2}^{ + } + x \cdot (1 - \mu_{2}^{ + } - \nu_{2}^{ - } )$. Then

$$\mathop f\nolimits_{{(\tilde{\alpha }_{1} \ge \tilde{\alpha }_{2} )}} \left( x \right)\, = \frac{{\max \;\left( {0,\;\alpha_{1}^{ + } (x) - \alpha_{1}^{ - } (x) + \alpha_{2}^{ + } (x) - \alpha_{2}^{ - } (x) - \max \left( {0\;,\;\;\alpha_{2}^{ + } (x) - \alpha_{1}^{ - } (x)} \right)\;} \right)}}{{\alpha_{1}^{ + } (x) - \alpha_{1}^{ - } (x) + \alpha_{2}^{ + } (x) - \alpha_{2}^{ - } (x)}}$$

Let $$\left\{ \begin{gathered} b = \mu_{1}^{ + } - \mu_{1}^{ - } + \mu_{2}^{ + } - \mu_{2}^{ - } \hfill \\ d = \mu_{2}^{ + } - \mu_{1}^{ - } \hfill \\ c = \mu_{1}^{ - } - \mu_{2}^{ + } + \nu_{1}^{ + } - \nu_{2}^{ - } \hfill \\ k = \mu_{1}^{ - } + \mu_{2}^{ - } - \mu_{1}^{ + } - \mu_{2}^{ + } + \nu_{1}^{ + } + \nu_{2}^{ + } - \nu_{1}^{ - } - \nu_{2}^{ - } \hfill \\ \end{gathered} \right.$$

Then

$$\mathop f\nolimits_{{(\tilde{\alpha }_{1} \ge \tilde{\alpha }_{2} )}} \left( x \right)\, = \frac{{\max \;\left( {0,\;b + k \cdot x - \max \left( {0\;,\;\;d + c \cdot x} \right)\;} \right)}}{b + k \cdot x}$$

Based on the idea of a definite integral, the mean $M(\tilde{\alpha }_{1} \ge \tilde{\alpha }_{2} )$ and variance $V(\tilde{\alpha }_{1} \ge \tilde{\alpha }_{2} )$ of the possibility degrees of IVIFVs $\tilde{\alpha }_{1} \ge \tilde{\alpha }_{2}$ are defined as follows:

10 $$M(\tilde{\alpha }_{1} \ge \tilde{\alpha }_{2} ) = \int_{0}^{1} {\;\mathop f\nolimits_{{(\tilde{\alpha }_{1} \ge \tilde{\alpha }_{2} )}} \;\left( x \right)} \;dx,$$

11 $$V(\tilde{\alpha }_{1} \ge \tilde{\alpha }_{2} ) = \sqrt {\int_{0}^{1} {\;\;\left[ {\mathop f\nolimits_{{(\tilde{\alpha }_{1} \ge \tilde{\alpha }_{2} )}} \left( x \right) - M(\tilde{\alpha }_{1} \ge \tilde{\alpha }_{2} )\;} \right]}^{2} \;dx} .$$

The mean and variance of the possibility degrees of IVIFVs $\tilde{\alpha }_{1} \ge \tilde{\alpha }_{2}$ defined by Definition 10 have the following desirable properties:

1. $$0 \le M(\tilde{\alpha }_{1} \ge \tilde{\alpha }_{2} ) \le 1$$, $$0 \le V(\tilde{\alpha }_{1} \ge \tilde{\alpha }_{2} ) \le 1$$.
2. $$M(\tilde{\alpha }_{1} \ge \tilde{\alpha }_{2} ) + M(\tilde{\alpha }_{2} \ge \tilde{\alpha }_{1} ) = 1$$, $$V(\tilde{\alpha }_{1} \ge \tilde{\alpha }_{2} ) = V(\tilde{\alpha }_{2} \ge \tilde{\alpha }_{1} )$$.

### Example 4.2

Assume the same data as that in Example 4.1, through the weights of attributes and the IIIFWAA aggregation operators in Eq. (9), the assessments of different attributes can be aggregated as follows:

$$IIIFWAA_{A1} = ([0.5359,0.6331],[0.2025,0.3669]),IIIFWAA_{A2} = \left( {\left[ {0.5296, \, 0.5922} \right],\left[ {0.2100,0.3030} \right]} \right)$$

$$IIIFWAA_{A3} = \left( {\left[ {0.5535,0.5974} \right],\left[ {0.1849,0.3649} \right]} \right),IIIFWAA_{A4} = \left( {\left[ {0.5563,0.5920} \right],\left[ {0.1978,0.3489} \right]} \right)$$

According to Eqs. (10) and (11), matrix M of the means and matrix V of the variances of the possibility degrees of IVIFVs can be calculated as follows:

$$M{ = }\left[ {\begin{array}{*{20}c} {0.5} & {0.4988} & {0.5044} & {0.5049} \\ {0.5012} & {0.5} & {0.4965} & {0.4972} \\ {0.4956} & {0.5035} & {0.5} & {0.5012} \\ {0.4951} & {0.5028} & {0.4989} & {0.5} \\ \end{array} } \right],V{ = }\left[ {\begin{array}{*{20}c} 0 & {0.0731} & {0.0254} & {0.0313} \\ {0.0731} & 0 & {0.0556} & {0.0557} \\ {0.0254} & {0.0556} & 0 & {0.0053} \\ {0.0312} & {0.0557} & {0.0053} & 0 \\ \end{array} } \right].$$

Through Eqs. (5) and (6), the evaluation values of the alternatives can be obtained as follows: r(A1) = 0.2592, r(A2) = 0.2385, r(A3) = 0.2491, and r(A4) = 0.245. Therefore, the alternatives are sorted according to score value, and the ordered result is $A1 \succ A3 \succ A4 \succ A2$. From the score values, we can see that the ranking orders of alternatives are not in accordance with the method as shown in Example 4.1. The major factor is related to the use of means and variances. The method in Example 4.2 considers the influence of the mean and variance .Thus, the evaluation results can better reflect the differences between the alternatives. Compared with Example 4.1, we can see that the comprehensive evaluation values of the four alternatives in Example 4.2 differ greatly from each other. The identification degree of the evaluation results is significantly improved, and it is easier to sort and select the best alternative than other methods. It ensures the validity and accuracy of the ranking order.

## A new method for MAGDM with IVIFVs

To improve the performance of IVIFV assessment and eliminate the drawbacks mentioned in the previous section, a new method for MAGDM with IVIFVs is proposed. An air combat training accuracy assessment example is presented to demonstrate the applicability of the proposed method. Meanwhile, comparative analyses with other methods are conducted to show the superiority of the proposed method.

### The decision-making procedure

In this section, we propose a new MAGDM method based on the proposed aggregation operator and the means and variances of the possibility degrees of IVIFVs. Assume one MAGDM problem. Let $A = \left\{ {A_{1} ,A_{2} , \cdots A_{m} } \right\}$ be a set of alternatives, let $C = \left\{ {C_{1} ,C_{2} , \cdots C_{n} } \right\}$ be a set of attributes and let $A^{k} = (\tilde{\alpha }_{ij}^{k} )_{m \times n}$ be the decision matrix given by the decision-maker (DM), where $\tilde{\alpha }_{ij}^{k} = ([\mu_{ij}^{{{ - }k}} ,\mu_{ij}^{ + k} ],[\nu_{ij}^{{{ - }k}} ,\nu_{ij}^{ + k} ])$ is the evaluation of alternative $A_{i}$ with respect to attribute $C_{j}$ given by the DM k, $1 \le i \le m$,$1 \le j \le n$ and $1 \le k \le K$. Let $d{ = }\left\{ {d_{1} ,d_{2} , \cdots d_{K} } \right\}$ be the weight vector of DMs, and $\omega_{j}^{k}$ be the weight of attribute $C_{j}$ given by DM k. In the following, we present the proposed MAGDM method:

*Step 1* Through the weights of attributes and the new aggregation operator in Eq. (9), the decision matrix of DMs can be aggregated as $D^{1} ,D^{2} , \cdots D^{K}$.

*Step 2* According to Eq. (10) and Eq. (11), the means $M^{k} = (m_{ij}^{k} )_{m \times m}$ and variances $V^{k} = (v_{ij}^{k} )_{m \times m}$ of the possibility degrees of IVIFVs $A_{{_{i} }}^{k} \ge A_{{_{j} }}^{k}$ can be computed, where $1 \le i \le m$,$1 \le j \le n$ and $1 \le k \le K$.

*Step 3* The comprehensive evaluation values $h_{i}^{k} = [h_{i}^{k - } ,h_{i}^{k + } ] \, \left( {i = 1,2, \ldots m, \, k = 1,2, \ldots K} \right)$ of each DM with respect to each alternative can be computed as follows:

12 $$h_{i}^{k - } = \frac{{\sum\nolimits_{j = 1}^{m} {m_{ij}^{k} } + \frac{m - 2}{2}}}{m(m - 1)} - \frac{{\sqrt {\sum\nolimits_{j = 1}^{m} {(v_{ij}^{k} )^{2} } } }}{m(m - 1)},h_{i}^{k + } = \frac{{\sum\nolimits_{j = 1}^{m} {m_{ij}^{k} } + \frac{m - 2}{2}}}{m(m - 1)} + \frac{{\sqrt {\sum\nolimits_{j = 1}^{m} {(v_{ij}^{k} )^{2} } } }}{m(m - 1)}.$$

*Step 4* The comprehensive evaluation values $Z(A_{i} )$ of alternatives $A_{i}$ can be computed as follows:

13 $$Z(A_{i} ) = \sum\limits_{j = 1}^{K} {h_{i}^{j} \cdot d_{j} } ,$$

where $d = (d_{1} ,d_{2} , \cdots d_{K} )$ is the weight vector of DMs.

*Step 5* Based on the obtained intervals $Z(A_{i} )$, the possibility degree using Eq. (5) can be calculated, and then the evaluation values of each alternative by using Eq. (6) can be obtained.

*Step 6* Rank the alternatives according to the decreasing values of $r(A_{i} )$. The alternative with the highest evaluation value is the most desirable alternative.

**Figure 1:**
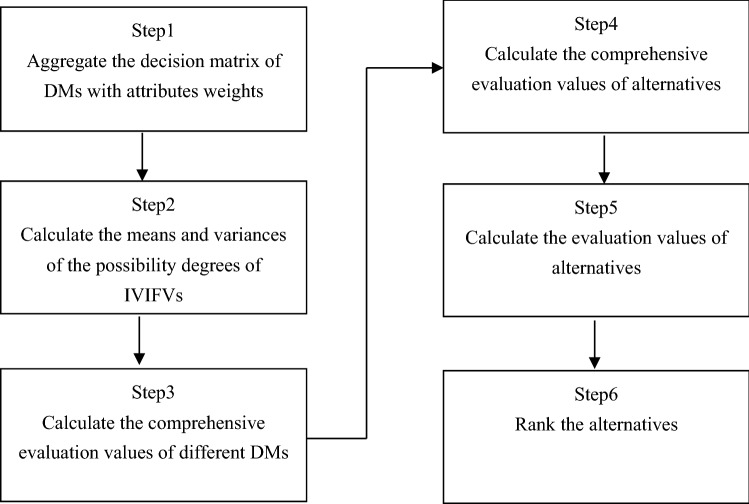
Flowchart of the proposed MAGDM method.

The decision making procedure of the proposed MAGDM method is summarized in Fig. 1.

In the following section, the proposed MAGDM method is applied to the air combat training accuracy assessment.

### Application example

To illustrate the applicability of the proposed method, we consider an example of an application from Hou et al.^21^ designed to evaluate hitting the target of an air combat training missile among four missiles A1, A2, A3,and A4. A total of four DMs participated in the evaluation of four missile alternatives by considering the following four evaluation criteria: miss distance C1, hit time C2, hit probability C3 and hit accuracy C4. The weight vector of the four DMs is d = [0.240, 0.265, 0.262, 0.233], and the weight vectors of the evaluation criteria are given in Table 1. Four experts in the field evaluate the alternatives based on their knowledge and experience, and use IVIFVs to express the evaluation value of each Ai (i = 1,…, 4) under Cj (j = 1,…,4), and the results are shown in Table 1. The proposed MAGDM method is employed to solve the assessment problem.

**Table 1:** Decision matrices of four DMs.

DMs	Missiles	C1	C2	C3	C4
DM1	A1	([0.7, 0.8], [0.1, 0.15])	([0.6, 0.75], [0.1, 0.15])	([0.6, 0.65], [0.15, 0.2])	([0.7, 0.8], [0.15, 0.15])
	A2	([0.7, 0.75], [0.15, 0.2])	([0.65, 0.8], [0.15, 0.2])	([0.65, 0.7], [0.25, 0.3])	([0.5, 0.55], [0.3, 0.35])
	A3	([0.8, 0.8], [0.1, 0.15])	([0.7, 0.8], [0.1, 0.15])	([0.7, 0.8], [0.1, 0.15])	([0.65, 0.7], [0.25, 0.3])
	A4	([0.6, 0.65], [0.25, 0.3])	([0.7, 0.8], [0.1, 0.15])	([0.7, 0.75], [0.15, 0.2])	([0.5, 0.55], [0.4, 0.45])
	Weights	0.22	0.17	0.25	0.36
DM2	A1	([0.7, 0.75], [0.15, 0.2])	([0.8, 0.8], [0.15, 0.2])	([0.7, 0.75], [0.15, 0.2])	([0.7, 0.75], [0.2, 0.25])
	A2	([0.75, 0.75], [0.15, 0.2])	([0.7, 0.75], [0.1, 0.2])	([0.65, 0.75], [0.1, 0.15])	([0.8, 0.8], [0.1, 0.2])
	A3	([0.65, 0.7], [0.15, 0.2])	([0.85, 0.9], [0.1, 0.1])	([0.6, 0.7], [0.25, 0.25])	([0.75, 0.75], [0.15, 0.2])
	A4	([0.75, 0.8], [0.15, 0.2])	([0.75, 0.8], [0.05, 0.1])	([0.55, 0.6], [0.35, 0.4])	([0.75, 0.8], [0.1, 0.15])
	Weights	0.19	0.18	0.22	0.41
DM3	A1	([0.65, 0.7], [0.2, 0.25])	([0.8, 0.85], [0.1, 0.15])	([0.8, 0.85], [0.1, 0.15])	([0.8, 0.85], [0.1, 0.15])
	A2	([0.8, 0.85], [0.1, 0.12])	([0.75, 0.85], [0.1, 0.15])	([0.75, 0.8], [0.15, 0.2])	([0.7, 0.8], [0.2, 0.2])
	A3	([0.8, 0.85], [0.1, 0.15])	([0.65, 0.65], [0.3, 0.35])	([0.7, 0.85], [0.1, 0.15])	([0.62, 0.65], [0.28, 0.3])
	A4	([0.85, 0.85], [0.1, 0.15])	([0.6, 0.75], [0.25, 0.25])	([0.73, 0.85], [0.12, 0.15])	([0.7, 0.7], [0.2, 0.25])
	Weights	0.21	0.19	0.23	0.37
DM4	A1	([0.6, 0.65], [0.25, 0.3])	([0.65, 0.7], [0.25, 0.3])	([0.65, 0.65], [0.25, 0.3])	([0.63, 0.65], [0.24, 0.3])
	A2	([0.7, 0.75], [0.2, 0.25])	([0.63, 0.65], [0.27, 0.3])	([0.58, 0.6], [0.2, 0.25])	([0.69, 0.73], [0.17, 0.25])
	A3	([0.59, 0.65], [0.21, 0.27])	([0.62, 0.65], [0.22, 0.35])	([0.6, 0.7], [0.23, 0.3])	([0.6, 0.65], [0.25, 0.3])
	A4	([0.8, 0.85], [0.1, 0.15])	([0.65, 0.65], [0.28, 0.35])	([0.67, 0.7], [0.25, 0.28])	([0.73, 0.75], [0.21, 0.25])
	Weights	0.24	0.18	0.21	0.37

The steps of the MAGDM procedure are explained as follows.

*Step 1 *The decision information on alternatives is aggregated with evaluation criteria weights by using the IIIFWAA aggregation operator described in Eq. (9). The aggregated IVIFV decision matrix is given in Table 2.

**Table 2:** Aggregated IVIFV decision matrix.

Missiles	DM 1	DM 2	DM 3	DM 4
A1	([0.6615, 0.7611], [0.1343, 0.1649])	([0.7211, 0.7598], [0.1747, 0.2402])	([0.7751, 0.8265], [0.1161, 0.1735])	([0.6311, 0.6596], [0.2383, 0.3404])
A2	([0.6153, 0.6887], [0.2268, 0.3113])	([0.7641, 0.7719], [0.1153, 0.2281])	([0.7448, 0.8217], [0.1829, 0.1783])	([0.6616, 0.6984], [0.2048, 0.3016])
A3	([0.7099, 0.7686], [0.1563, 0.2314])	([0.7304, 0.7716], [0.1595, 0.2284])	([0.6904, 0.7589], [0.2068, 0.2411])	([0.6013, 0.6612], [0.2318, 0.3388])
A4	([0.6159, 0.6950], [0.2480, 0.3050])	([0.7155, 0.7671], [0.1507, 0.2329])	([0.7326, 0.7864], [0.1649, 0.2136])	([0.7254, 0.7559], [0.2005, 0.2441])

*Step 2* Based on Eqs. (10) and (11), the mean and variance matrices of the probability degrees of four missiles corresponding to four DMs are calculated. Then the comprehensive values of the four DMs with respect to four missiles are obtained by Eq. (12), which are shown in Table 3.

**Table 3:** The comprehensive values of the four DMs with respect to four missiles.

Missiles	DM 1	DM 2	DM 3	DM 4
A1	[0.2630, 0.2929]	[0.2269, 0.2349]	[0.3414, 0.3622]	[0.1791, 0.1965]
A2	[0.1722, 0.1962]	[0.2683, 0.2775]	[0.2199, 0.2463]	[0.2503, 0.2772]
A3	[0.3620, 0.3781]	[0.2676, 0.2772]	[0.1771, 0.1947]	[0.1720, 0.1902]
A4	[0.1631, 0.1726]	[0.2180, 0.2295]	[0.2259, 0.2325]	[0.3596, 0.3752]

*Step 3* Consider the weight vector of four DMs d = [0.240, 0.265, 0.262, 0.233]. Based on Eq. (13), we obtain weighted comprehensive evaluation values of the alternatives in the form of interval numbers:

Z(A1) = [0.2544, 0.2732], Z(A2) = [0.2284, 0.2497], Z(A3) = [0.2443, 0.2595], Z(A4) = [0.2399, 0.2506].

*Step 4* Using Eqs. (5) and (6), the assessment scores of each alternative are calculated and given as follows:

$${\text{r}}\left( {{\text{A1}}} \right)\, = \,0.{3625},{\text{ r}}\left( {{\text{A2}}} \right)\, = \,0.{1629},{\text{ r}}\left( {{\text{A3}}} \right)\, = \,0.{2716},{\text{ r}}\left( {{\text{A4}}} \right)\, = \,0.{2}0{31}.$$

*Step 5* Finally, the alternatives are sorted in decreasing order according to r(Ai), and the ordered result is ${\text{A}}_{1} \succ A_{3} \succ A_{4} \succ A_{2}$.

### Comparative analysis and discussion

In this section, the performance of the proposed MAGDM method is verified by comparing it with the TOPSIS method and three other similar methods in the literature (Xu^30^, Yao^29^, Hou et al.^21^). The proposed method and the methods from the literatures are employed to perform the evaluation example^21^. Different methods are applied to the same decision-making problem and the same group of decision-making information to facilitate the comparative analysis of the results. The final order rankings of the four alternatives by applying these approaches are displayed in Table 4.

**Table 4:** Comparison of the evaluation results of different methods.

Methods	Assessment scores				Ranking order
	A1	A2	A3	A4	
Proposed method	0.625	0.1629	0.2716	0.2203	${\text{A}}_{1} \succ A_{3} \succ A_{4} \succ A_{2}$
Xu^30^	0.5531	0.5423	0.5199	0.5199	${\text{A}}_{1} \succ A_{2} \succ A_{3} { = }A_{4}$
Yao^29^	0.7741	0.7689	0.7576	0.7648	${\text{A}}_{1} \succ A_{2} \succ A_{4} \succ A_{3}$
TOPSIS	0.5736	0.4401	0.3833	0.4366	${\text{A}}_{1} \succ A_{2} \succ A_{4} \succ A_{3}$
Hou et al.^21^	0.7658	0.7568	0.7480	0.7553	${\text{A}}_{1} \succ A_{2} \succ A_{4} \succ A_{3}$

From Table 4, we can see that the ranking order is ${\text{A}}_{1} \succ A_{2} \succ A_{3} { = }A_{4}$ when using the method suggested by Xu^30^. Table 1 shows that A3 and A4 are two different alternatives, but the ranking order of A3 and A4 is A3 = A4. Therefore, the drawback of Xu’s method is that it cannot distinguish alternatives A3 and A4 in this case. In addition, it can be seen from Table 4 that the ranking orders of the alternatives obtained by the method of Hou et al.^21^ are the same as those obtained by Yao^29^ and those obtained by the TOPSIS method but not in accordance with those in the proposed method in this paper. The ranking order is obtained as $A_{1} \succ A_{2} \succ A_{4} \succ A_{3}$ for the methods suggested by Hou et al.^21^ and Yao^29^ as well as the TOPSIS method. However, the score values of the alternatives obtained by the method proposed by Yao^29^ and the TOPSIS method are all very close. In particular, the score value of A2 is very close to the value of A4. On the other hand, the ranking order is found as ${\text{A}}_{1} \succ A_{3} \succ A_{4} \succ A_{2}$ with the proposed IVIFV MAGDM method in this paper.

There are two important factors that lead to the ranking order differences. In the proposed method, the IIIFWAA operator takes the influence of the nonmembership degree in the interval-valued intuitionistic fuzzy information on the decision-making into account and places more emphasis on the role of individuals in the decision-making process. However, the IIFWA operator in the methods proposed by Xu^30^, Yao^29^ and Hou et al.^21^ emphasizes the role of the group, ignores the role of the individual in decision-making and is insensitive to the role of individuals. Therefore, the application of the IIFWA operator in decision-making will introduce decision error. In "Analysis of the drawbacks of IIFWA operators and the proposal of an improved IVIFV operator", it was shown that the use of the IIFWA operator may mislead the decision-making process if the lower bound of the nonmembership degree or upper bound of the nonmembership degree is zero. The second factor is related to the use of the matrices of the means and variances. The proposed method in this paper takes the influence of the means and variances of the possibility degrees of every alternative into account. Thus, the evaluation results can better reflect the differences between the alternatives. Compared with other methods, from Table 4, we can see that the comprehensive evaluation values of the four alternatives obtained with the proposed MAGDM method in this paper differ greatly from each other. The identification degree of the evaluation results is significantly improved, and it is easier to sort and select the best alternative using this method over the other methods. It ensures the validity and accuracy of the ranking order. As a result of these differences, the ranking order of the proposed MAGDM method was different than the that of the other methods.

On the basis of the above analyses, the proposed method has some advantages over other methods.

1. To aggregate the decisions of different DMs or attributes, some methods (Hou et al.^21^, Yao^29^ and Xu^30^) derived an aggregation decision matrix using the IIFWA aggregation operator of IVIFVs. When the nonmembership degree of an IVIFV is zero, the nonmembership degree of the IVIFVs aggregated by the IIFWA operator must be zero, even if the nonmembership degrees of other IVIFVs are not zero. Thus, the nonmembership degree of other IVIFVs cannot be reflected in the decision-making process. The IIFWA operator emphasizes the role of the group, ignores the role of individuals in decision-making and is insensitive to the role of individuals. As a result, the IIFWA aggregation operator will lose or distort the original decision information in the aggregation process. Therefore, the application of the IIFWA operator in decision-making will introduce decision error. In contrast, in this paper, the operational laws were redefined, and a new IVIFV aggregation operator of IVIFVs was proposed. The new aggregation operator takes the role of the nonmembership degree of each IVIFV into account in decision-making, reduces the loss or distortion of the original decision information in the process of aggregation and improves the sensitivity to individuals.
2. The use of the means and variances of the IVIFVs based on the concept of a definite integral was proposed to provide more comprehensive decision-making information for the decision-making process.
3. To rank the alternatives, in the method provided by Hou et al.^21^ and the TOPSIS method, the distances between the alternatives and the positive and negatives ideal solutions were calculated. Yao^29^ calculated the score values to rank alternatives. However, this ranking method cannot be used to distinguish some IVIFVs under some specific situations. When the attribute values of different alternatives are very close to each other, the final evaluation result of each alternative may be very close to one another or even equal. In this situation, the accuracy of the ranking order of alternatives remains to be discussed. In contrast, a new MAGDM method for IVIFVs was proposed in this paper. The proposed MAGDM method modified the comprehensive values by calculating the means and variances of the possibility degrees of IVIFVs. Therefore, the evaluation results can better reflect the difference between different alternatives, improve the identification of the evaluation results and make it easier to sort and select the best alternatives. Furthermore, it has some desirable properties and advantages over existing methods.
4. Some studies developed many useful MAGDM methods. When integrating DMs’ information, these methods assume that the weight of the same attribute given by different experts is the same. However, since decision-makers usually come from various research areas and there are many differences in their knowledge structure, expression abilities, evaluation levels, individual preference and practical experience, their corresponding opinions or evaluations may differ substantially. Therefore, the same attribute weights given by different DMs are different. In this paper, an approach to variable weight group decision-making based on the means and variances of the IVIFVs was proposed to better address uncertainty in the MAGDM problem.

## Conclusions, limitations, and future works

In this paper, we propose a novel MAGDM method based on the new IIIFWAA aggregation operator and the means and variances of the possibility degrees in an IVIFV environment. First, the novel operational laws and the new IIIFWAA aggregation operator are defined to ensure the effective communication of information overcome the drawbacks of the IIFWA aggregation operator and avoid losing or distorting the original decision information in the process of aggregation. Second, the means and variances of the possibility degrees of IVIFVs are defined and utilized to improve the identification of the evaluation results to alleviate the drawback that it is difficult to distinguish alternatives whose score values are close to each other. Third, a novel MAGDM method based on the IIIFWAA aggregation operator and the mean and variance of the possibility degrees of IVIFVs is proposed to ensure the effective communication of information among group members and improve the identification of the evaluation results. Finally, the proposed method is successfully applied to air combat training accuracy assessment. Moreover, a thorough comparison has been performed with related existing works to show the advantages and validity of the method.

The proposed method has the following advantages:

1. Less information loss. Since the new IVIFV aggregation operator takes the role of the nonmembership degree of each IVIFV into account in decision-making, and reduces the loss or distortion of the original decision information in the aggregation process, there is less loss of information when using this MAGDM method.
2. Comprehensive consideration. The MAGDM method considers not only the aggregation operator but also the means and variances of possibility degrees for each alternative. Therefore, its consideration is comprehensive.
3. Evaluation result identification improvement. The proposed method emphasizes the role of individuals in the decision-making process and is more sensitive to individuals. It considers the influence of the means and variances on the evaluation values. It also improves the identification of the evaluation results and makes the evaluation results more accurate and reliable.
4. Wide application. The MAGDM method has no limitation on the data distribution, the number of alternatives and the number of attributes. It is suitable for handling complex decision-making problems, including multiple DMs, multiple alternatives and multiple attributes.

Despite all the advantages, the developed MAGDM method still has some limitations. First, all initial decision-making information originated from the judgement of DMs based on their knowledge and experience, and the final result may be manipulated by experts, which will make the assessment results inconsistent with practical circumstances. In future work, we would like to establish a combination evaluation system that contains the machine learning method and expert assessment method, which can make the final results more reliable. Second, the weights of decision-makers are assigned as fuzzy numbers. Because experts usually come from various research areas and there are many differences in their knowledge structure, expression abilities, evaluation levels, individual preferences and practical experience, their opinions or evaluations may differ substantially. Thus, a consensus reaching process is necessary to reach a general consensus on the selected alternatives in MAGDM. In future work, we would like to study subjective assignment methods utilizing the IVIFS to assign the weights of different decision-makers to better address uncertainty in the MAGDM problem. Third, there are some other parameters, including the hesitancy degree, distance, etc., which could affect the decision results in the whole decision-making process. In the future, we will continue working on the mean–variance calculation method with hesitancy information, and combine it with the distance measurement method to extend the proposed method in this paper. Finally, the MAGDM method should be employed for other more practical problems under different environments to demonstrate its general flexibility and reliability. In future research, we will focus on increasing the adaptability of linguistic variables’ membership functions depending on the change in experts’ weight coefficients and should also focus on developing a trained artificial intelligence-based algorithm. We would like to extend the proposed method to the fields of management, environment, and so on.

## Supplementary Information

Supplementary Information.

## Data Availability

All data generated or analyzed during this study are included in this published article.
