# Supplementary material for: A multiattribute group decision-making method based on a new aggregation operator and the means and variances of interval-valued intuitionistic fuzzy values
Source: Sci Rep. 2022 Dec 29;12:22525. doi: 10.1038/s41598-022-27103-z (PMC9800407; doi:10.1038/s41598-022-27103-z)
Supplement: Supplementary file 1 — Supplementary Information. [file 41598_2022_27103_MOESM1_ESM.pdf]

Manuscript Number: 6a1f30d7-3aaf-4e97-93f5-c574ffa7b2af

Article title:

"A multi-attribute group decision making method based on new aggregation operator and means and variances of interval -valued intuitionistic fuzzy values"

Authors list:

Authors	Email address	Primary affiliation
Ruipu Yao (First Author)	yrptj@tjcu.edu.cn	Tianjin University of Commerce
Huijuan Guo (Corresponding Author)	guohuijuan@tjcu.edu.cn	Tianjin University of Commerce

Ruipu Yao conceived the study and Huijuan Guo collected the data. Ruipu Yao and Huijuan Guo wrote the main manuscript text and reviewed the manuscript.
